# Methods of Suicide Used by People With Cancer: A Scoping Review

**DOI:** 10.1002/pon.70476

**Published:** 2026-05-03

**Authors:** Doireann Ni Dhalaigh, Zubair Kabir, Fahmi Ismail, Paul Corcoran, Daisy Wiggin, Eugene Cassidy

**Affiliations:** ^1^ University College Cork Cork UK; ^2^ Cork University Hospital Cork UK; ^3^ National Suicide Research Foundation Cork UK

**Keywords:** access to means, cancer, scoping review, suicide, suicide methods

## Abstract

**Background:**

Individuals diagnosed with cancer are at increased risk of suicide compared to the general population. Access to means is a key volitional risk factor for suicide and represents a modifiable target for prevention at a population level. Understanding the methods of suicide in this group is crucial to inform targeted prevention strategies.

**Aims:**

This scoping review aimed to examine the methods of suicide used by individuals with cancer, identify the types of study designs used to investigate this topic, and evaluate the feasibility of a future systematic review.

**Methods:**

This scoping review followed JBI methodology and the PRISMA‐ScR checklist. A comprehensive search was conducted in five databases: Medline (EBSCO), PsycINFO (EBSCO), Embase (Elsevier), CINAHL Plus (EBSCO), and Web of Science (Clarivate) and gray literature. Studies were eligible if they quantitatively reported suicide methods in individuals with cancer. Studies of non‐fatal self‐harm, case reports, or those lacking method‐specific data were excluded.

**Results:**

Twenty‐seven studies from multiple countries were included. Method choice was influenced by geographical location, age, and sex. There was considerable heterogeneity in study design, cancer definitions, control groups used, and the characteristics of comparison samples reported.

**Conclusions:**

Due to substantial methodological variation, a systematic review is not currently feasible. Future research should, at a minimum, control for age and sex, include underrepresented populations, and adopt region‐specific approaches.

## 1 Introduction

Individuals with cancer are at a higher risk of suicide than those without cancer. Identified risk factors include male sex, older age, diagnosis within the previous 12 months, and certain cancer types [1]. Previous systematic reviews and meta‐analyses have largely focused on suicide mortality in cancer, with considerable variation by cancer type, such as pancreatic (SMR 6.42), bone and cartilage (SMR 9.59), and mesothelioma cancers (SMR 13.07). Suicidal ideation has received less attention, but increased risk has been reported in reviews of prostate and bladder cancers, including a twofold higher risk in prostate cancer in the first year post‐diagnosis [2].

The Three‐Step Theory (3ST) may help to understand why cancer populations are at increased risk. This model includes four main factors to explain suicide: pain, hopelessness, connectedness and capability [3]. It is acknowledged that medical conditions, such as cancer, can cause pain and hopelessness and can reduce connectedness via the inability to engage in social activities, thereby creating suicidal desire. Capability for suicide strongly influences whether someone with suicidal desire goes on to attempt suicide [3]. In cancer populations, the practical capability for suicide may be increased via access to potentially lethal means, such as medications used in cancer care. Access to lethal means is a key determinant of suicide risk, and restricting access is a well‐established prevention strategy [4, 5, 6, 7]. Cancer management may involve multiple medications, including opioids, which, if taken in overdose, increase its lethality [8]. While this access to medication may constitute an increased access to lethal means, the suicide methods used by individuals with cancer have not been systematically reviewed.

The method of suicide varies across countries, sex, and age. Hanging is the most common method in Europe, followed by drug poisoning and drowning [9]. In contrast, in the USA, firearms are the most common method of suicide, especially among older males and females [10]. In South Asia, hanging is followed by poisoning, often due to pesticides [11] Method use varies elsewhere in Asia, from hanging in Bahrain to poisoning in Bangladesh and China, and jumping from a height in Hong Kong [12].

Among adults aged ≥ 65, older men, especially in those aged > 80, most commonly die by hanging or firearms than those aged < 65. Older women are more likely to die by poisoning and jumping, whereas younger women more commonly use hanging and a broader range of more violent methods [13].

In summary, individuals with cancer face a higher risk of suicide compared to those without cancer and may have greater access to means of suicide. However, no review to date has specifically examined the available evidence on the methods of suicide used within this population. This scoping review will be used to identify if there is scope to conduct a systematic review of methods of suicide used by people with cancer in comparison to a control population, by mapping the literature, identifying knowledge gaps, and assessing heterogeneity.

This scoping review aimed to examine what is known about the methods of suicide used by people with cancer and was an initial step in assessing the feasibility of a systematic review. The objectives included identifying study designs, cancer classifications, method categorisations, and comparators.

## 2 Methods

A protocol for the review was published [14] and is reported in line with the PRISMA‐ScR guidelines [15] Checklist included as Supporting Information S1: Appendix A. This scoping review was conducted following JBI guidelines, using the Population, Concept(s), and Context (PCC) framework [16] [See Supporting Information S1: Appendix B].

### 2.1 Population Concept Context PCC

#### 2.1.1 Population

The population included individuals diagnosed with any type or stage of malignancy, including pre‐ or post‐mortem diagnoses, to encompass all relevant research due to the lack of prior scoping reviews on this topic.

#### 2.1.2 Concept

The concept is methods of suicide. This includes deaths of undetermined intent due to the challenges in proving intent due to a lack of evidence. This excludes non‐fatal self‐harm and/or suicide attempt. Due to international variation in suicide definitions [17], studies using any author‐ or country‐defined classification of suicide or probable suicide were included. To account for under‐reporting [18], deaths of undetermined intent were also included. Reported methods were extracted as presented. Studies on attempted suicide, accidental deaths, parasuicide, or non‐fatal self‐harm were excluded, as these were conceptually distinct.

#### 2.1.3 Context

Studies had to quantify suicide methods in cancer populations, excluding case studies and series without statistical representation. Psychological autopsy studies were included if the study reported quantitative data. See Table 1 for a summary of inclusion and exclusion criteria.

**TABLE 1:** Summary of inclusion and exclusion criteria.

Population			
Inclusion	Rationale	Exclusion	Rationale
Single or multiple cancer types	Broad coverage for cross‐cancer comparisons	Suicide methods are not reported separately for cancer	Unable to distinguish the methods used by individuals with cancer
Any cancer stage or remission status	Identifies distinctions between active cancer, remission, and survivorship	Cancer is grouped with other diseases	Unable to isolate suicide methods specific to cancer
Cancer diagnosis	Ensures the inclusion of individuals with cancer	Studies solely on physician‐assisted suicide (PAS) or euthanasia	Focused on a single method distinct from conventional suicide
Cancer survivor	Includes those with a current or past cancer diagnosis	Refusal of treatment	As above
Physician‐assisted suicide/PAS/euthanasia (reported as quantitative data as an additional method of suicide)	Included if quantified as a proportion of total suicides		
Concept			
Completed suicides	Focus on fatal outcomes	Non‐fatal self‐harm (attempts, parasuicide)	Focus on fatal outcomes
Probable suicides	Fatal outcome. Includes cases of likely suicides	Suicidal ideation	Non‐fatal outcome
Deaths of undetermined intent—where the specific method used is reported and quantified	Captures potential suicide cases. Potentially mitigates against misclassification of suicide deaths.	Suicide methods not quantified	Lack of quantifiable data
		Methods are only reported narratively	Lack of quantifiable data
			Case reports lack systematic reporting and quantifiable data.
Context			
Studies on all suicide methods used by a population or sample with cancer	Ensures proportions are determinable	Studies focused on a single method	Requires a different search strategy; future reviews may explore specific methods
No year restrictions	Ensures all relevant studies are captured	Non‐English studies	Resource constraints prohibit systematic inclusion
Studies without exact figures on methods	Includes studies with overall trends	Commentaries, letters to the editor	Lack of methodological rigor
Case series (reported quantitatively)	Quantifiable data	Case series (reported singularly or narratively)	Lack of quantifiable data
Studies on all suicide methods in cancer	A comprehensive understanding of the methods used	Studies focused on a single method	Requires a different search strategy; future reviews may explore specific methods
		Reviews	The focus is on primary research

### 2.2 Information Sources

The search strategy included a headings search consisting of two concepts: cancer and suicide, and a text search consisting of three concepts: cancer, suicide, and methods, combined using the Boolean operator AND. The full search can be found on the OSF: [19]. The following databases were searched: Medline (EBSCO), PsycINFO (EBSCO), Embase (Elsevier), CINAHL Plus (EBSCO), and Web of Science (Clarivate). The last search was performed on: 03.01.25. No restrictions on the publication date, and it was limited to the English language. Forward and backward citation searching, a gray literature, and a Google Scholar search were also performed. Following the screening and selection process, the data sources identified in the included papers (publicly available data repositories, government and health authority websites) were searched, using the search “cancer” AND “suicide”; “methods of suicide” AND “cancer.” Authors from the included papers were also contacted for additional sources. Three authors responded. Two provided additional papers, and one provided the anonymised study data [20].

### 2.3 Screening and Selection

The screening and selection processes outlined in the published scoping review protocol were followed [14]. An additional researcher joined the team to assist with additional screening requirements due to the updated search strategy recommended by peer review of the protocol.

### 2.4 Data Extraction and Protocol Amendments

The data extraction form was piloted independently by two reviewers (DND & DW). Quantitative data on suicide methods were extracted and charted separately by both reviewers. All other data were extracted by one reviewer (DND) and checked by another (DW). Given the absence of identified prior reviews in this area, the data extraction process was conducted iteratively. This was expected and detailed in the published protocol [14]. The data collection tool was amended during data extraction, and all changes are documented in the supplementary material.

All the data items are openly available on the Open Science Framework (OSF). For better clarity, these data items are reported on two separate Excel sheets in OSF; one for suicide methods [22], and one for information about the data source [23]. Methods of suicide have been reported as they are presented, and for cross‐study comparisons, these were then grouped into eight distinct categories [1], firearms [2], hanging/suffocation [3], jumping/falling [4], poisoning [5], drowning [6], cutting/stabbing/blunt force trauma [7], lying in front of a moving object, and [8] other (specified or unspecified). These were then subsumed into six categories by incorporating the two with the most N/A or zero entries [6]: cutting/stabbing/blunt force trauma and [7] lying in front of a moving object, into the ‘other’ category. Finally, for comparative purposes, two broader categories were created (poisoning vs. all other methods) to facilitate clearer cross‐study comparisons. For suicide methods, a narrative synthesis approach was used, due to variability in coding and terminology used for suicide methods. Similar methods were grouped in adjacent columns, and overarching category headings were applied. Each of these four spreadsheets (i.e., suicide methods as reported, eight, six, and two categories) is available on the OSF.

Data are presented graphically, narratively, and in tables where applicable. Descriptive statistics and graphical representations are used to illustrate findings concerning suicide methods.

## 3 Results

**FIGURE 1:**
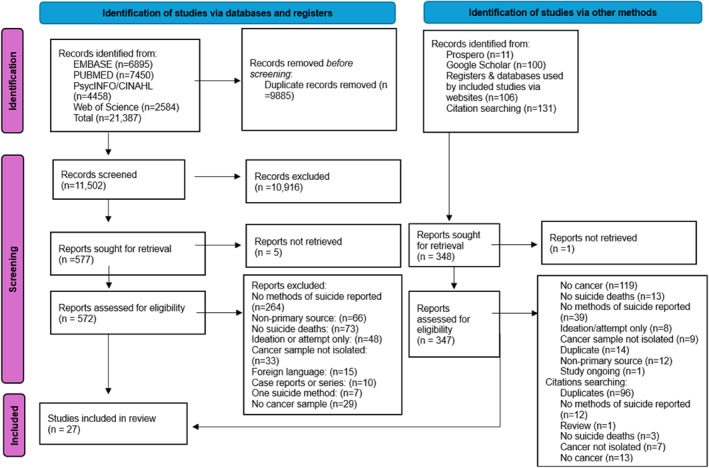
PRISMA flow diagram of screening and selection results [21].

As depicted in Figure 1 PRISMA flowchart, 27 papers were included following the screening and selection process. The papers spanned multiple regions including Europe; Scotland [24], Sweden [25, 26, 27, 28], Finland [29, 30], Norway [31], Switzerland [32], Austria [33], France [20], Italy [34], Poland [35], and Lithuania [36]; East Asia; Japan [37, 38, 39, 40], Taiwan [41, 42], and Hong Kong [43], USA [44, 45, 46, 47, 48], and New Zealand [49]. The sources of the data used are detailed in Table 2: Study Characteristics. The included studies employed a range of study designs. The majority (*n* = 17) were retrospective cohort studies. The remainder comprised four case‐control studies [20, 42, 47, 48], two psychological autopsy studies [25, 34], one prospective cohort study [37], and a root‐cause analysis study [44]. Two studies applied time‐series or trend analysis methodologies [32, 46]. Only three studies [29, 38, 41] explicitly identified examining suicide methods as a specific aim. Others used more ambiguous language, such as referring to the “circumstances of suicides” [48] or “characteristics of suicide” [34, 43], making it difficult to determine whether the analysis of suicide methods was a primary objective.

**TABLE 2:** Study characteristics.

Lead author and year of publication	Study design	Data sources & dates	Cancer criteria and case identification	Min & max age (suicide + cancer + methods reported)	Cancer types included	Cancer stages reported	Suicide methods by cancer subgroups
Aboumrad et al. (2018) [44]	Root‐cause analysis study	Veterans' health administration national center for patient safety root‐cause analysis (RCA) database: Cancer‐related suicide cases between 2002 and 2017, which records suicides that occur within 3 days of medical discharge or 7 days of psychiatric discharge	Suicides occurring during oncology care, including diagnosis, treatment, or during 5‐year follow‐up RCA reports defined death as “cancer‐related suicides” how this was established is unclear. Excluded suicides with insufficient RCA details, or patients more than 5 years post‐treatment	Not reported	Prostate, lung, colon, head and neck, lymphoma, leukemia, other	Yes: By stage in care (e.g., diagnosis, curative treatment, palliative treatment, follow‐up)	All male sample
Allebeck et al. (1989) [26]	Retrospective cohort	Swedish cancer‐environment register covering the period 1961–79 (which is regularly matched with cause of death register)	Cancer cases reported to the Swedish cancer register – Posthumous cancer diagnosis excluded	Not reported	Lip, tongue, mouth, pharynx, gastrointestinal, colon and rectum, upper airways and lungs, breast, prostate, genital organs, uterus, ovaries, kidney, skin, blood, lymphatic system	No	Methods by sex
Bolund (1985) [25]	Psychological autopsy	Death certificates, medical records, forensic medical records, and police reports from suicide cases with a diagnosis of cancer registered from 1973 to 1976.	Cancer diagnosis confirmed through medical or forensic records. – Excluded if cancer diagnosis was incorrect	40–80	Various cancer types, though specific types not reported	No	Methods by sex
Camidge et al. (2007) [24]	Retrospective cohort	SMR1 (morbidity records) linked with SMR6 (national cancer register) diagnosed with cancer between 1981 and 1995	– Cancer listed as a comorbidity on the death certificate. First diagnosed cancer only; invasive cancer only; Excluded: – Non‐melanoma skin cancer; – Posthumous cancer diagnosis excluded	Not reported	Respiratory, digestive tract, female genital tract, head and neck, digestive, respiratory, skin, lymphoid, secondaries	No	Methods by time since cancer diagnosis
Cheung et al. (2017) [49]	Retrospective cohort study	Coronial data: Coroner records of all older people (age 65+) who died by suicide in New Zealand during study period	– Terminal cancer only – Identified from: police reports, postmortem findings, and general practitioner reports. Min age 65 years	Min 65	Colorectal, lung, bladder, prostate, pancreas, liver, skin, mediastinum, vagina	Terminal only	No
Chung and Lin (2010) [41]	Retrospective cohort	National health insurance Research database (NHIRD) & cause of death data file provided by the taiwanese department of health (DOH) between 1 January 2002 and 3 1 December 2004	Patients hospitalised in Taiwan with a principal diagnosis of cancer (ED‐9‐CM codes 14O.XX239.m	Not reported	Oral, gastrointestinal, respiratory, breast, genitourinary, hematological, others	No	Violent versus non‐violent reported by: Urbanicity level Gender Age group Marital status Employment status Suicide area (in Taiwan) Monthly income category Type of malignancy Place of death (hometown versus out of town Charlson index score
de la Grandmaison (2014) [20]	Case‐control	All adult cases that underwent forensic autopsy from January 2002 to December 2012 in Western Paris: forensic autopsy reports	– Cancer in situ at autopsy cancer could be a major causative factor, or an incidental finding excluded bodies showing an advanced state of putrefaction were excluded from the study (autopsy study). Excluded cases only diagnosed post‐mortem.	Not reported	Thyroid, prostate, kidney, lung, liver, esophageal, breast	Yes: Presence of metastasis (Y/N)	No
Fujimori et al. (2017) [40]	Retrospective cohort	Suicide data (death certificates and supplementary documents) obtained from the Tokyo Medical Examiner's office from 2009 to 2013 and police interviews with bereaved families and hospitals/clinics where the deceased were treated.	– Any current or history of cancer. Identified using interviews with family/healthcare workers and medical records.	26–97	Stomach, colon, rectum, lung, prostate, breast, head and neck, liver, gynecologic, hematologic, bladder, esophageal, pancreatic, kidney, multiple primary cancers	No	Methods by sex and age (< 65, ≥ 65)
Gentile et al. (2022) [34]	Retrospective case‐series analysis	Database of the institute of forensic medicine in Milan, clinical records, family reports, police reports	– Cases identified based on clinical records and decedents suicide notes the – Excluded cases with dementia or unconfirmed/post‐mortem cancer diagnosis – Only subjects aware of their cancer diagnosis included	25–94	Head, neck, chest, abdomen, reproductive system, urinary system, immune system, skin, skeletal system	Yes: Existence of distant metastasis Y/N	Methods by sex and by tumor site
Guth et al. (2023) [32]	Time‐series analysis	Swiss federal statistical office (FSO) cause‐of‐death statistics & cancer registry database: Suicide cases from: 1999–2018.	– Cancer listed as comorbidity on death certificate Included any cases that listed malignant disease as a comorbidity on the death certificate from national death statistics data	Not reported	Prostate, breast, colorectal, lung	Not reported	No
Han et al. (2021) [46]	Time‐series analysis	Multiple cause of death database. CDC wide‐ranging ONline data for epidemiologic research (CDC WONDER) used to identify all suicides from 1999–2018	– Cancer listed as contributing cause to suicide death on death cert. Cancer‐related suicides were those with cancer as a contributing cause (ICD‐10 codes: C00‐C97 and D00‐ D09).	15+	Lung, prostate, colon, rectum, lymphoma, leukemia, head and neck, secondary sites, other cancers	No	No
Harashima et al. (2021) [39]	Retrospective cohort	National cancer registry in Japan & death certificate data, national statistics for general population mortality: Diagnosed between 1 January and 30 June 2016 and	Cancer cases meeting the criteria of intraepithelial and malignant tumors (ICD‐O‐3) and other specific conditions in the national cancer registry (NCR). – Patients diagnosed between January–June 2016 only included. – Posthumous diagnoses excluded – Uncertain diagnosis dates excluded. – Patients with CNS tumors were excluded from the cardiovascular death analysis. – For those with multiple cancer diagnoses, the most recent diagnosis date was used	Not reported	Stomach, colon, lung, prostate, rectum, esophagus, breast, pancreas, bladder, other	Yes: Localised, regional, metastatic, unknown/other	No
Hem et al. (2004) [31]	Retrospective cohort	All cancer patients registered in the cancer registry of Norway 1960 to 1997 linked to suicide diagnosis in the register of deaths at statistics Norway.	Cancer diagnoses registered from 1960–1997 – Excluded posthumous diagnoses – Excluded cancer that was on death certificate only with no further info available	Not reported	Buccal cavity, esophagus, stomach, colon, rectum, bronchus, lung, breast, female genital, prostate, urinary system, brain, skin, lymphatic, hematopoietic, others	Yes: Localised; nonlocalised; unknown	Methods by sex and decade (1960–1999)
Hietanen et al. (1994) [30]	Case control/Psychological autopsy	As part of a finish national suicide prevention project ‐all suicides that took place in Finland from 01.04.1987 to 31.03.1988 recorded by psychological autopsy method.	– Any current or history of cancer Any previous cancer identified from relatives or health‐care personnel and later confirmed by medical case records – Includes cancer as main underlying factor, a contributing factor or unrelated to suicide death	29–89	Gastrointestinal tract, head and neck, lung, prostate, lymphoma, breast, melanoma, pancreas, corpus uteri, other	In remission, terminal & active phase	No
Hultcrantz et al. (2015) [28]	Retrospective cohort	The Swedish cancer register was used to identify all individuals diagnosed with a hematological malignancy Between 1st January 1992 and 31st December 2006. Information on suicides was obtained from the cause of death register. Included all cases of suicide that occurred within 3 years of diagnosis.	– Hematological malignancies – Reported in the Swedish cancer register clinical details from patient medical records – First diagnosis of hematological malignancy during study period. – Excluded prior malignancies and patients under 18	Not reported	Hematological malignancies (multiple myeloma, non‐hodgkin lymphoma, acute myeloid leukemia, polycythemia vera)	Yes: Active treatment; including corticosteroids; in remission with no ongoing treatment; palliation	Methods by sex
Lin et al. (2009) [42]	Case‐control	National health insurance research dataset (NHIRD) & cause of death file (published by the department of health, Taiwan): 2002 to 2004	All cancer patients discharged from hospitals from 2002–2004 who died by suicide within 90 days of discharge – a Principal diagnosis of any type of neoplasm from icd codes in insurance database	Not reported	Oral, gastrointestinal, respiratory, breast, genitourinary, hematological, others	No	No
Lu et al. (2013) [27]	Retrospective cohort	All cases of cancer diagnosed between the ages of 15 and 30 in Sweden during 1987–2009. obtained from: Swedish population and housing censuses & nationwide cancer, causes of death, migration, and inpatient Registers	First primary cancer diagnosis registered on Swedish cancer registry – Cancer diagnosed during adolescence (15–30 years) – Excluded cases diagnosed through autopsy. First cancer diagnosis only age at diagnosis between 15 and 30 years	15–30	Testis, melanoma, brain, Hodgkin's, non‐Hodgkin's, cervix, thyroid, colon, rectum, breast, ovary	No	No
Massetti et al. (2018) [48]	Case‐control	CDC's national violent death reporting system (NVDRS) & law enforcement and coroner/medical examiner reports: 2004 to 2013.	– Any current or history of cancer Keywords in narratives indicating a cancer diagnosis or treatment history – Excluded ambiguous cases	Not reported.	Not reported	Not reported	No
Men et al. (2021) [43]	Retrospective cohort	Hong Kong Coroner's court suicide reports: 2003 and 2017	– Any current or history of cancer Narrative keywords from medical history and family interviews from Coroner's court reports. – “Cancer history” in medical reports	Not reported	Not reported	Not reported	No
Michalek et al. (2023) [35]	Retrospective cohort	Polish national cancer registry (PLCR) & nationwide death registry: Diagnosed with cancer between 2009 and 2019.	Identified cancer related ICD codes from cancer registry. – Only primary malignant neoplasms (the most recent diagnosis of a primary malignant neoplasm was included in the study of patients with two or more independent coexisting neoplasms). Excluded posthumous cancer diagnoses – Excluded non‐melanoma skin cancers.	Not reported	All cancer diagnoses listed in ICD‐10	No	Methods by sex
Miller et al. (2008) [47]	Case‐control	New Jersey pharmaceutical assistance program for the aged and disabled (PAAD) in New Jersey & medicare database & cause of death file from New Jersey department of vital Statistic – Health care utilization data from the year before the index date	Cases coded as malignancy: ICD code 140.xx‐208.xx in the year preceding death on death register	65+	Prostate, lung, colorectal, hematologic	Yes: Metastatic disease (Y/N)	No
Nugent et al. (2021) [45]	Retrospective cohort	Veterans' health administration data & US department of Défense's suicide data repository (populated by the national death index)	Head and neck cancer survivors diagnosed with stage I to IVB – Included only survivors expected to have longer survival: (Therefore excluded stage IVC, second primary cancer, HNC recurrence within 2 years, and salivary/thyroid cancers)	Not reported	Head and neck cancer survivors	Localized; non‐localised; unknown	No
Pukkila et al. (2000) [29]	Retrospective cohort	The population‐based, prospectively collected data sample consisted of 1515 completed suicides committed in the province of Oulu, Finland, during the period 1988–99. Data (official death certificates, which are based on medico‐legal investigations (autopsy reports included as part of the death certificates) from suicide deaths that occurred in Oulu, Finland, during the period 1988–99	– Cancer ICD code on death cert. Cancer was identified based on ICD‐9 and ICD‐10 diagnostic codes from death certificates.	37.9–79.5	Not reported	Not reported	No
Smailyte et al. (2013) [36]	Retrospective cohort	Lithuanian cancer registry and census data & death records and population register database: 2001–2009	Patients with invasive cancer diagnosed in Lithuania from cancer registry during the study period. – Only invasive cancer cases were included – First diagnosis only Posthumous cases excluded – Cases where date of death matching date of diagnosis excluded	Not reported	All malignant neoplasms (C00–C96) and ‘other sites of cancer'	No	Methods by sex
Tanaka et al. (1999) [38]	Retrospective cohort	Hospital‐based cancer registry of the Osaka medical center for cancer and cardiovascular diseases & inquiries to the local offices of justice & Osaka cancer registry: Between 1978 and 94.	All patients residing in Osaka from 1978–1994 at time of diagnosis – Unknown causes of death excluded – Individuals who moved outside Osaka prefecture were excluded. – > 15 years, resided in Osaka prefecture at the time of diagnosis, – Newly diagnosed only	15+	Stomach, colorectum, lung, genital organ, male genital organ, others	Yes: Localized, regional, distant, unknown	Methods by location of suicide (hospital vs. non‐hospital)
Vyssoki et al. (2015) [33]	Retrospective cohort	Austrian cancer registry & cause of death from statistics Austria	Present on cancer registry, one type of cancer only (per individual) – Excluded posthumous diagnoses – Excluded cases with cancer at two or more sites.	Not reported	Prostate, breast, colon, rectum, skin, lymphoma, leukemia, lung, CNS, oesophagus, liver, pancreas, testis, other	Yes: stage I: Localized; II: Early locally advanced; III: Late locally advanced; IV: Metastasized	No
Yamauchi et al. (2014) [37]	Prospective cohort	Japan public health center‐based prospective study (JPHC study) & death certificates and population‐based cancer registries from 1990–2010.	Identified through active notifications from hospitals, cancer registries, and supplementary death certificate data. – Cancer diagnosed during study period – First cancer diagnosis only: – Excluded. Current (at baseline) or history of cancer – > 40 years of age at diagnosis	Inclusion criteria: > 40 years	Not reported	Yes: Localized or regional/distant.	Methods by hazard (< 52 weeks) versus control period (< 352 weeks)

### 3.1 Study Characteristics

#### 3.1.1 Quality Appraisal

The included studies were assessed using JBI critical appraisal tool for case series [50], cohort and case‐control studies [51]. The results, scoring (expressed as % “yes”), and rationales are available on the OSF [52]. Although most studies were rated as having moderate or low risk of bias, the majority (*n* = 24) did not include suicide methods as a primary aim, limiting the applicability of the appraisal of this outcome. The quality appraisal, therefore, addresses the overall study validity, not suicide methods as an outcome. Only one study had “suicide method” (defined as violent suicide vs. non‐violent suicide method) as a primary outcome [41]. An “External Validity/Generalisability Note” was also added to the quality appraisal table. External validity varied considerably across studies. The strongest generalisability was observed in large, population‐based studies from northern, central, and eastern Europe [24, 26, 27, 28, 30, 33, 35, 36], as well as national studies from Japan [39], Taiwan [41], and Hong Kong [43]. However, some relied on older data from the 1960s–1990s [26], reducing the relevance to modern‐day settings.

#### 3.1.2 Heterogeneity in Cancer Samples

There was considerable variation in how cancer was defined, operationalised, and in the inclusion and exclusion criteria used. As detailed in Table 2, four studies (15.4%) included individuals with any cancer history [30, 40, 43, 48]. Two used autopsies to confirm cancer at death [20, 34]. Others required cancer to be listed as a contributing cause [46], comorbidity [24, 32], or as a medical diagnosis [25] on the death certificate. One study quantified the link between the cancer and suicide, where it was reported that cancer was the main cause in 62% of suicides, contributory in 23%, and unrelated to 15% of suicide cases [30]. Only one study included only individuals who were aware of their cancer diagnosis before death [34], and one study reported that only 6 of the total 20 participants were aware of their cancer diagnosis before death [20]. Others identified cases via presence on a national cancer registry [24, 26, 27, 28, 31, 33, 35, 36, 37, 39]; hospital‐based register [38]; insurance or patient safety databases [41, 42, 44, 45, 47].

Two studies focus on specific cancer types; head and neck cancer survivors [45] and hematological cancers [28]. The reminder included mixed cancer types, with some restricting inclusion to invasive cancers [24, 36] or terminal cancers [49]. Eleven studies [20, 28, 30, 31, 33, 34, 37, 38, 39, 44, 45] reported some form of cancer staging within their samples; however, most did not examine suicide methods in relation to or delineated by stage. Only two studies [34, 41] reported the proportions of suicide methods by cancer type, and only one study statistically examined the association between suicide method and cancer characteristics [41]. The study also reports that violent methods^1^ were more common among males, older adults, the unemployed, those with lower income, breast cancer, higher comorbidity, and deaths that took place in one's hometown. In addition, in adjusted analyses, violent methods were less likely with genitourinary cancer and higher income, and more likely when death occurred outside the hometown.

**FIGURE 2:**
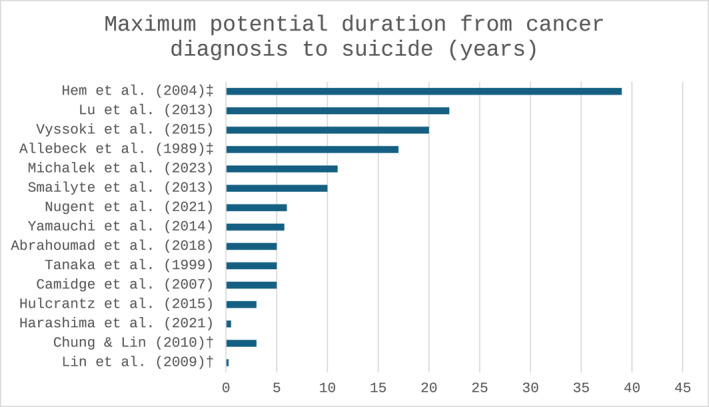
Maximum Calculated Duration from Cancer Diagnosis to Suicide (years). ‡ (If registry entry is an accurate proxy of date of diagnosis); † Since hospitalisation.

There were substantial differences in the time elapsed between cancer diagnosis and death by suicide, ranging from 6 months to 35 years, or as short as 90 days post‐hospitalisation (Figure 2). Four studies [30, 40, 43, 48] included individuals with any history of cancer, meaning some decedents may have been in remission long before their death. Time from diagnosis to suicide could only be estimated when the date of diagnosis was explicitly reported.

#### 3.1.3 Suicide Risk in Cancer Samples

Fifteen studies reported suicide risk estimates for cancer samples [20, 24, 26, 27, 28, 31, 33, 35, 36, 37, 38, 39, 42, 46, 47]. Risk estimates were reported using the following: standardised mortality ratios (SMR), odds ratios (OR), relative risk (RR), and hazard ratios (HR). Some included deaths of undetermined intent [24, 26]. One study examined changes in risk over time [46]. Estimates varied by time since diagnosis, by comparison group used, cancer criteria, type, and the inclusion criteria concerning the link between the cancer diagnosis and suicide.

### 3.2 Suicide Methods

#### 3.2.1 Suicide Method Classification

The classification of suicide methods varied by period and data source. Seven studies that used coroners' reports, autopsy findings, or death certificates [21, 25, 29, 30, 34, 40, 43] and the two studies that used veteran affairs data [44, 45] generally lacked standardised coding. In contrast, three larger registry‐based studies [24, 32, 46] used ICD coding systems available during the data collection period. One study used the National Violent Death Reporting System (NVDRS), a large, almost national dataset covering the majority of US states, described “suicide mechanisms”, which are based on ICD coding systems [48]. Other studies did not specify any coding criteria for suicide methods. Many studies grouped ICD categories into broader classifications, such as ‘poisoning’ or 'firearms', rather than reporting each ICD code individually. Authors generally did not provide a rationale for this approach; however, it is most likely due to sample size limitations, particularly for less common methods.

One study reported events of undetermined intent along with suicide methods [26]. In this case, therefore, these data are reported twice, once including events of undetermined intent and secondly excluding these. Another reported one event of undetermined intent, but did not report the specific method used [37].

Seven studies [28, 33, 37, 38, 39, 45] did not report the suicide methods of the entirety of their samples with cancer. Some [28, 37, 38], focused on higher‐risk periods following diagnosis. For example, one study reported the methods of suicide used by individuals who died within 3 years of diagnosis, only within their larger sample [28]. Another focused on the 5 years post‐diagnosis [38]. Others omitted reporting less common methods of suicide [33, 39, 45].

Many studies were further limited by small numbers of cancer‐related suicides or *n*=< 10 in suicide‐method subgroups [24, 26, 27, 28, 29, 31, 36, 37, 39, 49]. Other studies had narrow geographic scope, focusing on single cities or regions such as Milan [34], Tokyo [40], Paris [20], a single Japanese hospital [38], 10 Japanese public health centers [37], one Finnish province [29] or New Jersey [47]. Studies targeting specific populations, such as veterans [44, 45] and lower‐income older adults [47] were also limited in terms of generalisability. Overall, while some studies provide robust population‐level insights, others are dated, geographically narrow, or limited by small sample sizes.

#### 3.2.2 Geographic Variation

Suicide methods varied significantly by country. Hanging appeared to be the most consistently reported method overall (Table 3) and was particularly common in Poland [35], Lithuania [36], Japan [37, 38, 39, 40] and Hong Kong [43]. Firearms were predominant in the US [44, 45, 46, 47, 48], and to a lesser extent, Switzerland [32], and were seldom reported in Asian countries. The highest proportion of jump/fall from height was reported in studies based in Asia [27, 39, 40, 41, 43], followed by Milan in Italy [34]. Poisoning was most frequently reported in countries in Northwest Europe [24, 25, 26, 27, 28, 29, 30, 31, 32], New Zealand [49] and Taiwan [41, 42], except one study of US Veterans [45]. Some studies included small sample sizes (*n* = < 22) [20, 27, 47, 49]. These studies were also more likely to report higher rates categories under “other”.

**TABLE 3:** methods listed by geography ‐ 8 categories.

	Firearm	Hanging/suffocation	Jump/Fall from height	Poisoning	Drowning	Intentional self‐harm by moving object	Sharp or blunt instrument	Other (specified and unspecified)	Total n	Country
Aboumrad et al. [44]	75.0%	5.0%	n/a	n/a	n/a	n/a	5.0%	15.0%	65	USA
Allebeck et al. [26]	13.1%	33.7%	7.3%	30.5%	10.8%	n/a	2.7%	1.9%	849	Sweden
Allebeck et al. (inc. Undetermined deaths) [26]	11.6%	29.8%	7.1%	35.5%	11.0%	n/a	2.4%	2.6%	963	Sweden
Bolund [25]	15.9%	26.1%	11.4%	37.5%	5.7%	n/a	2.3%	1.1%	88	Sweden
Camidge et al. [24]	5.3%	16.8%	9.9%	45.1%	14.5%	n/a	4.6%	3.8%	131	Scotland
Cheung et al. [49]	39.1%	34.8%	n/a	17.4%	n/a	n/a	n/a	8.7%	23	New Zealand
Chung and lin [41]	n/a	38.3%	14.1%	37.2%	4.9%	n/a	2.9%	2.6%	1065	Taiwan
de la Grandmaison et al. [20]	50.0%	25.0%	15.0%	n/a	n/a	n/a	5.0%	5.0%	20	France
Fujimori et al. [40]	n/a	63.2%	18.4%	9.5%	3.2%	3.6%	n/a	2.2%	506	Japan
Gentile et al. [34]	18.4%	14.1%	51.2%	8.1%	2.8%	2.8%	2.5%	n/a	283	Italy
Guth et al. [32]	34.4%	20.5%	13.5%	19.7%	n/a	2.2%	n/a	9.7%	832	Switzerland
Han et al. [46]	72.7%	9.0%	n/a	14.0%	n/a	n/a	n/a	4.3%	6487	USA
Harashima et al. [39]	n/r	73.1%	11.0%	4.8%	n/r	n/r	n/r	n/r	129	Japan
Hem et al. [31]	22.2%	28.2%	8.8%	23.3%	11.9%	n/a	3.9%	1.7%	589	Norway
Hietanen et al. [30]	23.0%	47.0%	2.0%	16.0%	3.0%	n/a	n/a	9.0%	60	Finland
Hultcrantz et al. [28]	11.1%	27.8%	16.7%	22.2%	13.9%	n/a	n/a	8.3%	54	Sweden
Lin et al. [42]	n/a	36.1%	17.0%	36.3%	4.8%	n/a	2.9%	2.9%	311	Taiwan
Lu et al. [27]	n/a	45.5%	9.1%	36.3%	9.1%	n/a	0.0%	0.0%	22	Sweden
Massetti et al. (unmatched cancer cohort) [48]	71.2%	9.8%	n/a	14.4%	n/a	n/a	1.9%	2.7%	4182	USA
Men et al. [43]	n/a	27.5%	54.3%	9.1%	4.6%	n/a	n/a	4.5%	1461	China
Michalek et al. [35]	1.9%	84.6%	4.9%	4.1%	0.9%	0.5%	2.5%	0.7%	830	Poland
Miller et al. [47]	63.0%	15.0%	n/a	11.0%	n/a	n/a	n/a	11.0%	19	USA
Nugent et al. [45]	70.2%	n/r	n/r	29.8%	n/r	n/r	n/r	n/r	47	USA
Pukkila et al. [29]	30.3%	21.2%	n/a	30.3%	n/a	n/a	n/a	18.2%	33	Finland
Smailyte et al. [36]	5.6%	81.9%	3.7%	3.7%	1.9%	n/a	2.7%	0.5%	215	Lithuania
Tanaka et al. [38]	n/a	51.3%	28.2%	5.1%	5.1%	n/a	2.6%	7.7%	39	Japan
Vyssoki et al. [33]	25%	42%	13%	n/a	n/a	n/a	n/a	20%	33	Austria
Yamauchi et al. [37]	n/a	71.0%	9.7%	12.9%	n/a	n/a	n/a	6.5%	31	Japan

*Note:* A color gradient was used to indicate the differences in percentage values (darker red = higher percentage).

#### 3.2.3 Poisoning

As outlined in the scoping review protocol, people with cancer are often prescribed multiple medications with a high case fatality when taken in overdose, potentially increasing access to a specific method of suicide [14]. Therefore, evidence on substance‐specific poisoning was mapped in depth. Ten studies [24, 25, 30, 35, 40, 41, 42, 46, 47, 49] reported details of specific substances used in poisoning suicides, which are detailed in Supporting Information S1: Appendix C. The substances used varied considerably across studies, but in general, drugs were more commonly reported in studies from Northwest Europe and Taiwan, and to a lesser extent in the US. As previously outlined, poisoning deaths were most reported in these countries. Three studies specifically reported no suicides attributable to cancer‐related medications [30, 34, 47], while others reported no drug overdose deaths in cancer cohorts [38].

Poisoning was more often used by females than by males. Descriptive findings indicate that the greater the overall proportion of poisoning deaths, the larger the male/female gap (see graph included as Supporting Information S1: Appendix D). The younger the mean age at death, the higher the proportion of poisoning deaths (see heatmap included as Supporting Information S1: Appendix E). There also appears to be a relationship between the proportion of males in study samples and the proportion of non‐poisoning suicide methods (or more lethal methods) used (see graph included as Supporting Information S1: Appendix F).

#### 3.2.4 Suicide Method Distributions by Groups

##### 3.2.4.1 Suicide Methods ‐ Comparison Groups With Cancer

One study used a case‐crossover design to compare methods within 1 year of diagnosis versus later [37]. However, small method‐specific sample size limits meaningful interpretation. One study compared suicide methods to suicide attempt methods by individuals with cancer [27] and another reported suicide methods used by individuals with cancer, externally caused injuries, and deaths due to undetermined intent [39].

##### 3.2.4.2 Suicide Methods—Comparison Groups Without Cancer

Seven studies compared cancer and non‐cancer groups from broader cohorts [20, 29, 30, 40, 43, 48, 49]. Five studies compared cancer patients to the general population [24, 25, 26, 32, 46]. Some further stratified non‐cancer cases by contributing factors [29]. In others, the cancer group (*n* = 19) was included in the comparator sample (*n* = 128) of older adults with other medical conditions [47]. Sample sizes ranged from 60 to over 738,000. Five of these studies provided descriptive comparisons [20, 24, 25, 26, 29]. Two of these studies [24, 25] report fewer hanging deaths among individuals with cancer and one reported more hanging suicides in individuals with cancer [26]. Two studies report more poisoning suicides among individuals with cancer [25, 29] and one less poisoning suicides in individuals with cancer [26].

##### 3.2.4.3 Suicide Methods—Statistical Analysis

Eight studies conducted statistical analyses on suicide methods [30, 32, 40, 41, 43, 46, 48, 49]. In one study, unadjusted statistical analyses showed higher firearm use in cancer suicides (34.4% vs. 23.2%, *p* < 0.001) [32], while another found a trend toward more violent methods in terminal cancer cases (63.6% vs. 59.5%, ns; *n* = 23) [49]. Both studies noted that cancer groups were older and had more males. Four studies adjusted for age and sex [30, 40, 43, 48], and results were mixed. Two studies report higher use of violent methods among cancer patients, particularly older individuals [43, 48], and two found no significant differences [30, 40]. The level of adjustment for confounders varied across studies, with some controlling for sex and age and others including additional confounders (as outlined in Supporting Information S1: Appendix G).

#### 3.2.5 Trends Over Time in Suicide Methods

One study reported a decline in firearm‐related suicides among cancer patients (AAPC = −3.7%), with no significant trends for other methods [46]. Another study noted a decrease in poisoning from 1999 to 2008 [32].

#### 3.2.6 Time Since Diagnosis and Suicide Methods

Only two studies reported suicide methods according to time since diagnosis. One study used a case‐crossover design to describe suicide methods by time since diagnosis [37], and another reported proportions of suicide method use across different time intervals post‐diagnosis [24]; however, no statistical analyses were conducted. As a result, conclusions regarding the relationship between time since diagnosis and suicide method remain limited.

## 4 Discussion

### 4.1 Summary of Findings

This review identified considerable variation in how cancer was defined and operationalised across studies reporting suicide methods. Suicide methods varied by cancer type, sex, age, income, and geographic region. Poisoning was more commonly used by females. Poisoning was also more prevalent in both sexes in studies from Northern Europe and Taiwan. Older age and male sex were generally associated with more violent methods. However, the methodological differences and population variability in these studies should be considered when interpreting these findings. Two studies that adjusted for age and sex at a minimum found that individuals with a current or history of cancer were more likely to use violent methods compared to non‐cancer controls. However, each study adjusted for additional confounders that were not comparable.

Substance‐specific reporting was limited. Where these data were provided, a wide range of substances were identified, and the level of granularity varied. Drug‐related poisonings were more common in studies reporting a higher proportion of poisoning deaths overall. In the few studies that specified the drug involved, cancer‐related or analgesic medications did not predominate. Moreover, some studies in this review specifically reported no suicides attributable to cancer‐related medications. Some hypothesise that the reasons for this might be effective pharmacovigilance and restricted use of opioids (in Italy, in comparison to the US or Northern Europe) [34].

Only one study statistically analyzed the method choice by cancer type, where genitourinary cancers were associated with a higher likelihood of using non‐violent methods compared to respiratory cancers. The authors argue that this may be a reflection of differences in distress or treatment burden [41]. It has been noted that, generally, the more severe the cancer and the higher the baseline risk, the greater the impact on suicidality. Therefore, the short‐term risk of suicide associated with cancer may be understood using the Stress‐Diathesis Model of Suicide Risk for Patients with Cancer [53]. Perhaps, it may be possible to extend this model to suicide methods, given that there is evidence of increased use of more violent methods such as hanging, firearms, and the greatest risk is in those with a higher baseline risk (older males). For some individuals, cancer may act to exacerbate those most at risk using the most high‐risk methods; however, further research is needed. Interestingly, although one study reports that cancer type influenced suicide method choice, the same study reported that Charlson's index score levels, used as a proxy for severity, did not [41]. However, this could be due to the limitations of the measurement tool, as it is designed to measure comorbidities as opposed to cancer severity.

The descriptive synthesis in this scoping review indicates that the suicide methods used by cancer populations may vary according to age, sex, and country of origin, factors that also influence method choice in the general population. However, because few studies adequately controlled for these variables, and due to significant heterogeneity in cancer definitions, measurement, and follow‐up, it remains unclear how much these factors influence method choice compared to the disease itself. There was also considerable heterogeneity in the confounders adjusted for, preventing meaningful comparisons across studies and limiting the feasibility of a systematic review to address this question.

Some studies in this review suggested that the tendency toward older age and the use of more lethal methods may be more pronounced in cancer cohorts than in those without cancer. This indicates that further research may be valuable, as subgroup differences may not be apparent in descriptive analyses alone. This is particularly relevant for females, where the rarity of suicide limits statistical power for subgroup analyses. While the use of more lethal suicide methods may indicate that older males with cancer have a stronger intent to die; however, this may not reflect higher intent [54, 55]. While method choice affects the likelihood of death, sex and age also play a role; men have higher case fatality ratios (CFRs) than women, and CFRs increase with age [56]. Firearms have the highest CFR (89.7%), followed by hanging (84.5%), drowning (80.4%), gas poisoning (56.6%), jumping (46.7%), poisoning (8.0%), and cutting (4.0%) [57]. Drug poisoning CFRs vary by substance and age, with older adults showing higher rates. Male sex, older age, and multiple drug use are key predictors of fatal overdose [8]. In cancer patients, CFRs may be elevated due to poor health, age, and comorbidities.

Restricting access to means is one of the most effective evidence‐based methods to reduce suicide [7]. However, many violent suicide methods, such as hanging, are particularly difficult to control access to means. There is also the risk of substitution. Individuals may be more likely to switch between violent methods, such as firearms and hanging, than from a non‐violent method, like poisoning, to a violent one [58]. Consequently, restricting access to poisoning, therefore, while less lethal than firearms or hanging, may offer more opportunities for intervention. Commonly used methods can change according to advances in technology and infrastructure, and include age and sex variations [10]. This means that restricting access to certain means will likely only benefit specific sub‐populations, such as the countries identified in this review with higher rates of poisoning deaths. In terms of restricting access to cancer‐related or psychiatric medications, careful consideration is required to ensure individuals are not harmed by any restrictions, such as increased pain, anxiety, depression, or reduced autonomy, which could paradoxically increase suicide risk. A systematic review of means restriction of poison found that reductions in poisoning suicides were not associated with increases in suicides using other methods [59]. However, this has not been investigated in populations with cancer. Drawing on Joiner's (2005) Interpersonal Theory of Suicide, it is conceivable that individuals habituated to pain, such as some cancer patients, may be more likely to use more violent suicide methods due to acquired fearlessness of death and pain. This could make method substitution, the clinical and individual variability observed in populations with cancer notwithstanding, potentially more likely in this cohort.

### 4.2 Limitations

There are several limitations to this review that may affect the interpretation and generalisability of findings. These include methodological constraints, heterogeneity in how cancer‐suicide relationships were defined, potential code shifting, small sample sizes, variation in assisted suicide legality, and lack of data on time since diagnosis.

Two reviewers independently extracted the data on suicide methods; however, the remaining data extraction was conducted by a single reviewer and checked by a second, which may reduce the consistency or accuracy of the extracted information. However, as previously outlined, all extracted data is available on the OSF. Additionally, due to limited resources, non‐English language records were not included, as the team could not ensure comprehensive coverage of all relevant non‐English sources. No studies in this review were from lower‐ or middle‐income countries, which could be due to this limited search strategy.

There was substantial variation in how studies linked cancer to suicide, with cancer classified as a comorbidity, contributing cause, or general diagnosis, reflecting the array of study designs and data sources. Some studies required only that the decedent was on a cancer registry; few considered remission status, and none used it as a censoring criterion. As outlined in this review, one study included only individuals who were aware of their cancer diagnosis prior to death, while another reported that only 6 out of 20 individuals knew about their diagnosis before death. One study highlighted the potential effect of awareness of diagnosis as inclusion criteria due to low disclosure rates in Japan [37]. Large epidemiological studies can lack important contextual data about circumstances at the time of suicide [32]. Being labeled a “cancer patient” does not necessarily mean the individual was actively experiencing the disease or was distressed by it at the time of death.

Code shifting could lead to misclassification of intentional drug overdoses as accidental suicides, to reduce stigma, or due to uncertainty of intent [24]. One study in this review reported cases of undetermined intent along with suicide methods, and the majority were poisoning. Another study highlighted underreporting of drug use in suicides involving multiple methods, where drowning followed an overdose [25]. Moreover, that study also highlighted that analgesics were not examined as part of the forensic autopsy routine at the time of data collection. While these practices have most likely improved since that study took place, in 1985, (based on data from 1973–1976), international variation in investigation standards may persist.

Several studies had small sample sizes, particularly those reporting males and females separately, where female sample sizes were especially limited once subcategorised by suicide method. To address this, some studies, and this review, grouped methods into broad categories such as violent and non‐violent. However, this approach could lead to overly broad generalisations moreover, there is no consensus on what constitutes a violent method and what constitutes a non‐violent. While most studies classed all methods except poisoning as non‐violent [41, 43], others included drowning as a non‐violent method also [34].

Assisted suicide was illegal during data collection in most studies; however, there were exceptions. For the studies based in the US, assisted suicide was legal in some states, but not in others, making it challenging to determine whether it impacted the results. In Switzerland, where assisted suicide is legal, Guth et al. (2023) argue that the stability in poisoning rates in conventional suicide, compared to the increase in assisted suicides and the different proportions of males and females in conventional suicide versus assisted suicide, may indicate a distinct population [32].

Few studies considered time since diagnosis, despite its relevance to suicide risk [60], and potentially access to means. One study observed a shift toward pharmaceutical methods over time since cancer diagnosis, which is argued may reflect greater access to medications as the disease progresses [24]. However, this may also reflect survivorship bias, as individuals using more violent methods may not survive to later follow‐up periods. No statistical tests were conducted, and follow‐up intervals were unequal. The heightened suicide risk shortly after diagnosis and variation in cancer prognosis may influence suicide methods, but this remains unclear. However, as previously outlined, some cancer types have been associated with non‐violent methods [41].

### 4.3 Research Implications

Particular attention should be paid to low‐ and middle‐income countries (LMICs), which were absent in this review. While this may partly reflect the language restrictions applied, it may also reflect a lack of cancer suicide data from lower‐income countries [61]. Some initiatives aim to establish an international research network to investigate the risk of suicide in LMICs by sharing knowledge and expertise to link cancer registry and mortality data in LMICs [62]. Examination of suicide methods from mortality data should also be promoted.

Future studies should provide more granular details of the substances used in intentional poisoning deaths, where possible, via supplementary materials or secure repositories. This would facilitate examination of access to specific means of suicide. Future reviews may benefit from focusing on specific suicide methods or conducting qualitative syntheses of method‐related findings to deepen understanding. Hawton et al. 2024 advise that to assess the impact of any means restrictions, the specific segments of the population that are most likely to be affected by the restriction should be known. Poisoning was used more frequently by females and by younger age cohorts in this review however, this should be examined in greater detail before specific recommendations for means restriction can be given. It does, however open a potential avenue to examine in greater detail.

Suicide risk in cancer populations was examined to assess the feasibility of conducting a systematic review. Such a review would aim to determine whether differences in suicide risk could be linked to variations in the availability of suicide methods. However, due to the heterogeneity in risk estimate measures, cancer samples, and comparison groups found in this scoping exercise, a systematic review does not appear feasible at this time. This review underscores the importance of maximising homogeneity in cancer and chronological factors when studying suicide in cancer populations; however, achieving such homogeneity at present remains challenging due to limited research and the large sample sizes required for examining different suicide methods. Further studies are needed that, at a minimum, control for age, sex, and access to means or appropriate proxies before a systematic analysis can provide any robust results.

## 5 Conclusion

Restricting access to means is a key suicide prevention strategy at both the policy and practice levels. Understanding the methods used by individuals with cancer and subgroups within is essential to inform these strategies. Method choice is multifactorial, influenced by age, sex, location of death, country, income, and type of cancer. There are likely many more cancer‐related and access to means factors that influence suicide methods; however, small sample sizes have limited the ability to draw meaningful conclusions in some studies. Suicide among individuals with cancer is a rare occurrence, and dividing samples by suicide method, cancer type, or severity requires large sample sizes. This review found substantial heterogeneity in both individual characteristics and methodological approaches in studies that examined suicide methods used by populations with cancer. Cancer is a heterogeneous group of diseases, with considerable variation according to staging and/or metastasis. National cancer registries are a valid and representative method of case identification; however, they cannot alone measure how much, if any, influence that cancer had on that individual's suicide and suicide method. At present, it is unclear whether greater access to cancer medications increases access to suicide means at a population level. Further investigation of this method is needed among females, younger age cohorts and in specific regions.

## Conflicts of Interest

The authors declare no conflicts of interest.

## Supporting information


Supporting Information S1

## Data Availability

The data that support the findings of this study are openly available in Open Science Framework at https://osf.io/tvpb9/.

## Endnotes

^1^
Violent methods included: *ICD‐9‐CM* codes E953‐E958, including hanging, drowning, firearms, air guns and explosives, cutting and piercing instruments, jumping from high places, and other and unspecified. Means. Non‐violent methods: ICD‐9‐CM codes E950‐E952, including poisoning by solid or liquid substances, poisoning by gas used domestically, and poisoning by other gases and vapors.
