# Supplementary material for: Methods of Suicide Used by People With Cancer: A Scoping Review
Source: Psychooncology. 2026 May 3;35:e70476. doi: 10.1002/pon.70476 (PMC13136553; doi:10.1002/pon.70476)
Supplement: Supplementary file 1 — Supporting Information S1 [file PON-35-e70476-s001.docx]

## Appendix A: PRISMA-ScR Checklist:

Preferred Reporting Items for Systematic reviews and Meta-Analyses extension for Scoping Reviews (PRISMA-ScR) Checklist

| **SECTION** | **ITEM** | **PRISMA-ScR CHECKLIST ITEM** | **REPORTED ON PAGE #** |
| --- | --- | --- | --- |
| **TITLE** | | | |
| Title | 1 | Identify the report as a scoping review. | 1 |
| **ABSTRACT** | | | |
| Structured summary | 2 | Provide a structured summary that includes (as applicable): background, objectives, eligibility criteria, sources of evidence, charting methods, results, and conclusions that relate to the review questions and objectives. | 1 |
| **INTRODUCTION** | | | |
| Rationale | 3 | Describe the rationale for the review in the context of what is already known. Explain why the review questions/objectives lend themselves to a scoping review approach. | 3 |
| Objectives | 4 | Provide an explicit statement of the questions and objectives being addressed with reference to their key elements (e.g., population or participants, concepts, and context) or other relevant key elements used to conceptualize the review questions and/or objectives. | 4 |
| **METHODS** | | | |
| Protocol and registration | 5 | Indicate whether a review protocol exists; state if and where it can be accessed (e.g., a Web address); and if available, provide registration information, including the registration number. | 4 |
| Eligibility criteria | 6 | Specify characteristics of the sources of evidence used as eligibility criteria (e.g., years considered, language, and publication status), and provide a rationale. | 4 |
| Information sources* | 7 | Describe all information sources in the search (e.g., databases with dates of coverage and contact with authors to identify additional sources), as well as the date the most recent search was executed. | 5 |
| Search | 8 | Present the full electronic search strategy for at least 1 database, including any limits used, such that it could be repeated. | 5 |
| Selection of sources of evidence† | 9 | State the process for selecting sources of evidence (i.e., screening and eligibility) included in the scoping review. | 6 |
| Data charting process‡ | 10 | Describe the methods of charting data from the included sources of evidence (e.g., calibrated forms or forms that have been tested by the team before their use, and whether data charting was done independently or in duplicate) and any processes for obtaining and confirming data from investigators. | 6 |
| Data items | 11 | List and define all variables for which data were sought and any assumptions and simplifications made. | 6 |
| Critical appraisal of individual sources of evidence§ | 12 | If done, provide a rationale for conducting a critical appraisal of included sources of evidence; describe the methods used and how this information was used in any data synthesis (if appropriate). | n/a |
| Synthesis of results | 13 | Describe the methods of handling and summarizing the data that were charted. | 6 |
| **RESULTS** | | | |
| Selection of sources of evidence | 14 | Give numbers of sources of evidence screened, assessed for eligibility, and included in the review, with reasons for exclusions at each stage, ideally using a flow diagram. | 8 |
| Characteristics of sources of evidence | 15 | For each source of evidence, present characteristics for which data were charted and provide the citations. | 14 |
| Critical appraisal within sources of evidence | 16 | If done, present data on critical appraisal of included sources of evidence (see item 12). | n/a |
| Results of individual sources of evidence | 17 | For each included source of evidence, present the relevant data that were charted that relate to the review questions and objectives. | 6 |
| Synthesis of results | 18 | Summarize and/or present the charting results as they relate to the review questions and objectives. | 6 |
| **DISCUSSION** | | | |
| Summary of evidence | 19 | Summarize the main results (including an overview of concepts, themes, and types of evidence available), link to the review questions and objectives, and consider the relevance to key groups. | 26 |
| Limitations | 20 | Discuss the limitations of the scoping review process. | 27 |
| Conclusions | 21 | Provide a general interpretation of the results with respect to the review questions and objectives, as well as potential implications and/or next steps. | 28 |
| **FUNDING** | | | |
| Funding | 22 | Describe sources of funding for the included sources of evidence, as well as sources of funding for the scoping review. Describe the role of the funders of the scoping review. | 1 |

JBI = Joanna Briggs Institute; PRISMA-ScR = Preferred Reporting Items for Systematic reviews and Meta-Analyses extension for Scoping Reviews.

* Where *sources of evidence* (see second footnote) are compiled from, such as bibliographic databases, social media platforms, and Web sites.

† A more inclusive/heterogeneous term used to account for the different types of evidence or data sources (e.g., quantitative and/or qualitative research, expert opinion, and policy documents) that may be eligible in a scoping review as opposed to only studies. This is not to be confused with *information sources* (see first footnote).

‡ The frameworks by Arksey and O’Malley (6) and Levac and colleagues (7) and the JBI guidance (4, 5) refer to the process of data extraction in a scoping review as data charting*.*

§ The process of systematically examining research evidence to assess its validity, results, and relevance before using it to inform a decision. This term is used for items 12 and 19 instead of "risk of bias" (which is more applicable to systematic reviews of interventions) to include and acknowledge the various sources of evidence that may be used in a scoping review (e.g., quantitative and/or qualitative research, expert opinion, and policy document).

*From:* Tricco AC, Lillie E, Zarin W, O'Brien KK, Colquhoun H, Levac D, et al. PRISMA Extension for Scoping Reviews (PRISMAScR): Checklist and Explanation. Ann Intern Med. 2018;169:467–473. [doi: 10.7326/M18-0850](http://annals.org/aim/fullarticle/2700389/prisma-extension-scoping-reviews-prisma-scr-checklist-explanation).

## Appendix B: Population, Concept(s), and Context (PCC) framework (19)

| PCC Component | Description |
| --- | --- |
| Population | The population will be people with a diagnosis of any malignancy including single specific cancer types, multiple  malignancies, and samples that include a mix of cancer types.  This includes any stage, or remission status. This may be diagnosed pre or postmortem and may or not have been known to the decedent. These broad criteria have been chosen to facilitate the inclusion of all research on this population as no scoping reviews have been previously performed. |
| Concept | The concept is methods of suicide. This dictates that the participants in question must have died by suicide upon study  completion. Differences in nomenclature and definitions of suicide and suicide methods can exist internationally therefore  this review will include studies that use any definition of suicide or probable suicide as defined by the authors/country of publication. It is a possible limitation that cultural differences may lead to under-reporting of suicide and suicide methods in some countries, and this is true for all collations of international suicide data. In some countries, where there is insufficient evidence to decide if a death due to self-injury was intentional, for example in the case of an overdose, these are classed as deaths of undetermined intent. As the ultimate outcome is death in both the case of an accidental or intentional act of self-harm, and due to the challenges faced by coroner’s courts in deciphering intent in cases where no suicide note is found, this review will also include deaths of undetermined intent. Suicide methods will be extracted as reported. This is pertinent as no time limits will be imposed on the studies included. The ICD definitions of methods of suicide have changed significantly since its inception. For example, the ICD 8 includes 7 different categories of suicide methods whereas the ICD 11 includes 83 entries under intentional self-harm under parent category of external causes of morbidity and mortality and this, in addition to differences in data sources such as coroner’s reports or psychological autopsy studies, is likely to influence the range and nomenclature of reported suicide methods (Stewart *et al.*, 2017; WHO, 2024).  Attempted suicide, parasuicide, or any self-harm where the outcome is not fatal are outside the scope of this review as these conceptually differ from suicide methods that result in death. |
| Context | Studies had to quantify suicide methods in cancer populations, excluding case studies and series without statistical representation. Psychological autopsy studies were included if the study reported quantitative data. |

## Appendix C: Detailed Breakdown of Poisoning Suicides as Reported in Studies

| Study | Poisoning total *n* | Poisoning total % | Substances as % of total suicides |  |
| --- | --- | --- | --- | --- |
| Michalek et al. 2023 males | **25** | **3.68%** | X60 - nonopioid analgesics, antipyretics, and antirheumatics | 0.00% |
|  |  |  | X61 - antiepileptic, sedative-hypnotic, anti-parkinsonism, and psychotropic drugs | 0.26% |
|  |  |  | X64 - other and unspecified drugs, medicaments, and biological substances | 0.00% |
|  |  |  | X65 - alcohol | 3.16% |
|  |  |  | X67 – CO & other gases and vapours | 0.13% |
|  |  |  | X69 - other and unspecified chemicals and noxious substances | 0.13% |
| Michalek et al. 2023 females | **11** | **7.52%** | X60 - nonopioid analgesics, antipyretics, and antirheumatics | 0.63% |
|  |  |  | X61 - antiepileptic, sedative-hypnotic, anti-parkinsonism, and psychotropic drugs | 0.63% |
|  |  |  | X64 - other and unspecified drugs, medicaments, and biological substances | 3.75% |
|  |  |  | X65 - alcohol | 1.88% |
|  |  |  | X67 – CO & other gases and vapours | 0.63% |
|  |  |  | X69 - other and unspecified chemicals and noxious substances | 0.00% |
| Gentile et al. 2022 | **23** | **8.10%** | Antidepressants | 3.20% |
|  |  |  | Anxiolytics | 1.10% |
|  |  |  | Insulin | 0.70% |
|  |  |  | Acid caustic substances | 1.10% |
|  |  |  | Carbon monoxide (CO) | 2.00% |
| Han et al. 2021 (1999-2018) | **915** | **14.10%** | Drug-related | 10.90% |
|  |  |  | Chemicals and noxious substances | 3.20% |
| Men et al. 2021 | **133** | **9.10%** | CO2 poisoning | 6.60% |
|  |  |  | Other poisoning | 2.50% |
| Fujimori et al. 2017 males <65 | **12** | **10.25%** | Chemical and injurious materials | 5.98% |
|  |  |  | Substances | 4.27% |
| Fujimori et al. 2017 males >65 | **23** | **9.63%** | Chemical and injurious materials | 6.70% |
|  |  |  | Substances | 2.93% |
| Fujimori et al. 2017 females <65 | **7** | **9.72%** | Chemical and injurious materials | 8.33% |
|  |  |  | Substances | 1.39% |
| Fujimori et al. 2017 females >65 | **8** | **8.00%** | Chemical and injurious materials | 2.67% |
|  |  |  | Substances | 5.33% |
| Hultcrantz et al 2015 males | **5** | **26.20%** | Intoxication with sedatives/analgesics | 26.20% |
| Hultcrantz et al 2015 females | **3** | **23.10%** | Intoxication with sedatives/analgesics | 23.10% |
| Chung and Lin 2010 | **396** | **37.20%** | Poisoning by solid or liquid substances | 28.80% |
|  |  |  | Poisoning by gases in domestic use | 0.20% |
|  |  |  | Poisoning by other gases and vapours | 8.20% |
| Lin et al. 2009 (Adolescents) | **113** | **36.30%** | Poisoning by solid or liquid substances | 30.20% |
|  |  |  | Poisoning by other gases and vapours | 6.10% |
| Miller et al. 2008 | **2** | **11.00%** | Poisoning by drugs | 11.00% |
| Camidge et al. 2007 | **59** | **45.10%** | Analgesics, antipyretics, anti-rheumatics | 13.00% |
|  |  |  | Tranquillizers and psychotropics | 9.20% |
|  |  |  | Other substances | 9.90% |
|  |  |  | Gases | 13.00% |
| Hietanen et al. 1994 | **10** | **16.00%** | Drugs | 8.00% |
|  |  |  | Inhaling carbon monoxide | 8.00% |
| Tanaka et al 1999 | **2** | **5.12%** | Drug overdose (E950) | 0.00% |
|  |  |  | Poisoning of gas for house (E951) | 2.56% |
|  |  |  | Poisoning of gas for others (E952) | 2.56% |
| Bolund 1985 males | **17** | **28.80%** | Poisoning by solid or liquid drugs | 18.60% |
|  |  |  | Poisoning by gas | 10.20% |
| Bolund 1985 females | **16** | **55.20%** | Poisoning by solid or liquid drugs | 55.20% |
|  |  |  | Poisoning by gas | 0.00% |

## Appendix D: Poisoning vs all other methods of suicide in males and females with cancer

Poisoning vs all other methods of suicide in males and females with cancer

†including undetermined deaths

## AppendixE: Heatmap of poisoning versus all other methods of suicide listed by age at death.

|  | **Poisoning** | **All other methods** | **Mean age at death** | **Total *n*** |
| --- | --- | --- | --- | --- |
| Miller et al 2008 | 11% | 90% | 79 | 19 |
| Fujimori et al 2017 | 10% | 90% | 69 | 503 |
| Gentile et al 2022 | 8% | 92% | 66.6 | 283 |
| Men et al 2021 | 9% | 91% | 65.7 | 1461 |
| Massetti et al 2018* | 14% | 86% | 65.4 | 4182 |
| Hietanen et al 1994 | 16% | 84% | 63 | 60 |
| Pukilla et al 2000 | 30% | 70% | 59.4 | 33 |
| Chung and Lin 2010 | 37% | 63% | 59 | 1065 |

*Unmatched cohort

Heatmap of poisoning versus all other methods of suicide listed by age at death.

## Appendix F: Relationship between the proportion of males in study samples and the proportion of non-poisoning suicide methods used

Relationship between the proportion of males in study samples and the proportion of non-poisoning suicide methods used

## Appendix G: Results from studies that statistically analysed suicide method used by individuals/populations with cancer

| Lead author and year | Analysis conducted | Controls | Key findings |
| --- | --- | --- | --- |
| Analysed trends in cancer population over time | | | |
| Han et al 2021 | Join point regression analysis of age-adjusted trends in suicide methods over time (firearm, drug-related, chemicals/noxious substances, strangulation/suffocation/drowning/submersion) | not explicitly reported | Firearm-related suicides among cancer patients significantly declined (AAPC = -3.7%, 95% CI = -4.5% to -2.8%) but remained the most common method. No significant trend changes for hanging, suffocation, drowning, or drug-related suicides. |
|  |  |  |  |
| Compared individuals with cancer who used violent methods to non-violent methods | | | |
| Chung and Lin (2010) unadjusted analyses | Violent vs non-violent suicide methods |  | Those who used violent methods were more likely to be male, older, unemployed, to have low monthly incomes, breast cancer, to die in their hometowns, and to have higher Charlson’s index scores. |
| Chung and Lin 2010 adjusted analyses | aOR for violent vs. non-violent suicide methods in cancer patients, adjusting for demographic and clinical factors | Age, gender, marital status, employment, income, urbanisation, geographic region, place of death, malignancy type, Charlson Index (physical comorbidities) | Type of malignancy, monthly income, and place of death were significantly associated with the method of suicide even after controlling for related factors; genitourinary cancer patients were significantly less likely to use violent methods than those with respiratory cancer (OR = 0.55, p = 0.047). Higher income linked to lower violent method use (OR = 0.70, p = 0.042). Suicide outside one’s hometown increased likelihood of violent methods (OR = 1.39, p = 0.015). |
| Comparison of individuals with cancer to a control or reference group | | | |
| Cheung et al 2017 | Chi-square test comparing violent vs non-violent suicide methods between terminal cancer and non-terminal cancer groups | None | no statistically significant differences between violent & non-violent methods (n=23) |
| Guth et al | Chi-square test comparing methods used in cancer-associated conventional suicides to non-cancer-  associated  cases | None | During the 20-year observation period, shooting was the most frequent method to commit cancer-associated conventional suicides (34.4% vs. 23.2% compared to non-cancer-associated cases, p < 0.001. |
| Hietanen et al 1994 | Fisher’s exact test comparing suicide methods between cancer patients and controls | Sex and age matched control group | No significant difference in suicide methods between individuals with and without cancer |
|  |  |  |  |
| Fujimori et al 2017 | Chi-square test comparing suicide methods between cancer and cancer-free groups and across age groups | stratified by age (<65/≥65) and sex (male/female) | No significant differences in suicide methods between cancer and cancer free cases in males or females |
|  |  |  |  |
| Massetti et al 2018 | aOR for firearm, poisoning, suffocation, sharp instruments, and other methods in decedents with cancer history vs decedents with no cancer history | Matched on sex, age, race/ethnicity, year of death, marital status, and state of death | Firearm use was significantly higher among cancer decedents (71.9% vs. 65.3%, OR 1.35, 95% CI: 1.17–1.56). Suffocation was less common (OR 0.75, 95% CI: 0.62–0.90). Minor difference in poisoning prevalence. |
|  |  |  |  |
| Men et al 2021 | Multiple logistic regression for aOR of violent suicide methods (all methods excluding poisoning). | Adjusted for significant predictors (age, gender, marital status, employment, housing, living alone, suicide location, psychiatric comorbidities, physical conditions, prior suicidal intent). Age-stratified analysis included. | Cancer patients more likely to use violent methods than non-cancer cases (OR 1.7, 95% CI = 1.3–2.1), particularly in middle-aged (OR 1.6, 95% CI: 1.1–2.2) and elderly groups (OR 1.6, 95% CI: 1.0–2.4). |
| aOR (Adjusted Odds Ratio) | | | |
